# The Association of Metacognitive Beliefs With Emotional Distress After Diagnosis of Cancer

**DOI:** 10.1037/hea0000096

**Published:** 2014-08-18

**Authors:** Sharon A. Cook, Peter Salmon, Graham Dunn, Chris Holcombe, Philip Cornford, Peter Fisher

**Affiliations:** 1 Department of Psychological Sciences, University of Liverpool, and Royal Liverpool and Broadgreen University Hospitals NHS Trust, Liverpool, UK; 2 Centre for Biostatistics, The University of Manchester; 3 Royal Liverpool and Broadgreen University Hospitals NHS Trust; 4 Department of Psychological Sciences, University of Liverpool, Liverpool, UK

**Keywords:** cancer, anxiety, depression, PTSD symptoms, metacognitive beliefs

## Abstract

***Objective:*** Emotional distress after a diagnosis of cancer is normal and, for most people, will diminish over time. However, a significant minority of patients with cancer experience persistent or recurrent symptoms of emotional distress for which they need help. A model developed in mental health, the self-regulatory executive function model (S-REF), specifies that maladaptive metacognitive beliefs and processes, including persistent worry, are key to understanding why such emotional problems persist. This cross-sectional study explored, for the first, time whether metacognitive beliefs were associated with emotional distress in a cancer population, and whether this relationship was mediated by worry, as predicted by the S-REF model. ***Method:*** Two hundred twenty-nine participants within 3 months of diagnosis of, and before treatment for, primary breast or prostate cancer completed self-report questionnaires measuring anxiety, depression, posttraumatic stress disorder (PTSD) symptoms, metacognitive beliefs, worry, and illness perceptions. ***Results:*** Regression analysis showed that metacognitive beliefs were associated with symptoms of anxiety, depression, and PTSD, and explained additional variance in these outcomes after controlling for age, gender, and illness perceptions. Structural equation modeling was consistent with cross-sectional hypotheses derived from the theory that metacognitive beliefs cause and maintain distress both directly and indirectly by driving worry. ***Conclusions:*** The findings provide promising first evidence that the S-REF model may be usefully applied in cancer. Further study is required to establish the predictive and clinical utility of these findings.

Emotional distress is a normal response after cancer that, for most people, resolves spontaneously over time (Brennan, 2004; Salmon, 2000). However, for some, it persists for years after treatment (Burgess et al., 2005; Helgeson, Snyder, & Seltman, 2004). For instance, annual prevalence of major depression or generalized anxiety disorder remains 22% in the fourth year after breast cancer diagnosis (Burgess et al., 2005), and lifetime prevalence of cancer-related posttraumatic stress disorder (PTSD) is 10% to 12% for breast cancer and 20% for other cancers (Andrykowski & Kangas, 2010).

In recognition of this continuing psychological impact, health policies recommend that all patients undergo systematic psychological assessment at key points from diagnosis and have prompt access to psychological support (Holland, 1999; Institute of Medicine, 2007; National Institute for Health and Clinical Excellence, 2004). However, specialist help is limited and few patients have access to it. Most psychological care that is provided is offered reactively, that is, at the time of emotional crisis (Zabora et al., 2001). Moreover, there is little evidence that available psychological treatments are effective, with only small controlled effects sizes for anxiety (0.24) and depression (0.26) found in a recent meta-analysis (Naaman, Radwan, Fergusson, & Johnson, 2009) of breast cancer studies with high internal validity. Given that approximately 20% of patients experience clinically significant distress at some point in their cancer journey, a more cost-effective and ethical approach would be to identify the psychological processes that cause persistent distress so that a targeted preventative intervention can be provided.

Current theoretical approaches to understanding the causes of persistent distress after cancer share a common basis within the “cognitive paradigm,” that is, the view that distress is maintained by the individual’s negative appraisal of the illness. Initially, much of the research using these approaches was aimed at identifying specific coping strategies thought to mediate this relationship. However, this body of research yielded little of practical benefit (Somerfield, 1997). Consequently, research moved away from focusing on coping alone, toward understanding individuals’ cognitive representation of their cancer, in particular, the role of illness perceptions (comprising individuals’ thoughts, ideas, and beliefs about their illness) using the framework of Leventhal’s common-sense model (CSM) of self-regulation in health and illness (Leventhal, Nerenz, & Steele, 1984). Cross-sectional studies have confirmed associations between various illness perception dimensions and psychological outcomes in cancer (e.g., Dempster et al., 2012; Millar, Purushotham, McLatchie, George, & Murray, 2005; Rozema, Vollink, & Lechner, 2009; Scharloo et al., 2010; Traeger et al., 2009). However, a causal role for illness perceptions has yet to be demonstrated.

The fact that illness perceptions are not causally implicated in emotional distress is understandable, as most individuals receiving a diagnosis of cancer will experience some negative thoughts about cancer, yet not everyone experiences persistent distress. Negative thoughts are generally fleeting, and individuals’ thoughts about their cancer in the early stages are likely to be unstable as the individual is assailed with new information and experiences. Such illness-related thoughts only become a problem if the individual responds to them by engaging in excessive worry. Thus, it is probably not the illness perceptions per se, but the selection and use of worry in response to the negative thoughts that they trigger, which leads to persistent emotional distress. Worry is prevalent in cancer and, although a certain level is considered normal and adaptive, individuals who experience high levels of generalized worry are more like to feel helpless in response to their concerns (Parle, Jones, & Maguire, 1996) and to develop more negative illness perceptions (Lehto & Cimprich, 2009). However, cognitive models such as the CSM do not elaborate what causes such persistent worry.

An alternative and potentially more useful approach to understanding both what drives worry and what causes emotional distress after cancer is provided by the transdiagnostic self-regulatory executive function model (S-REF; Wells & Mathews, 1994). For most people, periods of distress in relation to cancer, or any other stressor, are transitory. However, the S-REF model proposes that distress becomes persistent when stored metacognitive beliefs guide the individual to select a particularly toxic style of sustained and inflexible conscious processing, known as the cognitive attentional syndrome (CAS). This includes cognitive processes such as persistent worry, focusing of attention on threat, and maladaptive coping strategies (e.g., avoidance or thought suppression). Continuation of the CAS is central to the development and maintenance of emotional disorder, although, as in traditional cognitive theory, it is dysfunctional beliefs that are considered key as to why it persists. However, in contrast to traditional cognitive theory, the S-REF model proposes that it is metacognitive beliefs, rather than the specific content of beliefs, about cancer that are important. Thus, the model proposes that activation and persistence of worry are fuelled by two types of metacognitive belief: positive beliefs about the benefits of, and need to engage in, worry (e.g., “If I worry about recurrence, I’ll detect early signs or symptoms”), which activate it; and negative beliefs about the danger or uncontrollability of worry (e.g., “I can’t stop worrying about my cancer returning”), which maintain and exacerbate it. According to this model, negative thoughts about cancer may activate metacognitive beliefs and worry, or may be a product of worry, but do not directly cause or maintain distress.

Challenging metacognitive beliefs and modifying components of the CAS have been used successfully to treat depression and a range of anxiety disorders in mental health settings (see Wells, 2009, for a review). In addition, metacognitive beliefs have been associated with heightened emotional distress in two physical health conditions: Parkinson’s disease (Allott, Wells, Morrison, & Walker, 2005) and chronic fatigue (Maher-Edwards, Fernie, Murphy, Nikcevic, & Spada, 2012). However, the utility of this model for explaining distress after cancer has not yet been explored.

The S-REF model predicts that negative illness perceptions will be associated with increased emotional distress, as has already been shown. However, due to the putative causal role of metacognitive beliefs about worry in activating and exacerbating the CAS in response to such cognitions, the S-REF model makes two new predictions. First, the model predicts that metacognitive beliefs will be able to explain additional variance in emotional distress, over and above that explained by negative illness perceptions. Second, it predicts that the relationship between metacognitive beliefs and emotional distress will be mediated by worry. Specifically, positive metacognitive beliefs will cause emotional distress by activating worry, but will have no direct effect, whereas negative metacognitive beliefs will maintain emotional distress both by triggering a direct emotional response and through exacerbating worry, including worry about worrying (metaworry).

This study aims to test these predictions by examining, for the first time, the relative contribution of negative illness perceptions and metacognitive beliefs to emotional distress after diagnosis of cancer and by testing the mediational role of worry.

## Method

### Participants

Participants were patients at least 18 years old attending routine pretreatment clinics at a National Health Service teaching hospital, after receiving a diagnosis of primary non metastatic breast or prostate cancer. Patients were excluded if they had recurrent or metastatic disease, or were considered by the clinical team or researcher to be too distressed or confused to give informed consent. The study was approved by the National Health Service North West 5 Research Ethics Committee (reference: 09/H1010/70).

Of 370 patients who were invited to participate, 258 (70%) consented and 229 (62% of those approached, 89% of consenters) returned completed questionnaires. There were no significant differences in age, gender, and tumor stage between consenting patients who returned completed questionnaires and those who did not.

### Measures

Emotional distress was measured using the Hospital Anxiety and Depression Scale (HADS; Zigmond & Snaith, 1983) and the Impact of Events Scale (Horowitz, Wilner, & Alvarez, 1979). The HADS is a well-established measure specifically developed to assess anxiety and depression in physically ill populations. Fourteen items are scored on a 4-point scale yielding two subscale scores of 0 to 21, with a cutoff score of 8 or more indicating a clinically significant level of anxiety or depression. The HADS has been extensively validated for use in cancer (Moorey et al., 1991; Vodermaier & Millman, 2011). The Impact of Events Scale is a 15-item self-report scale developed to assess the subjective impact of any specific event (e.g., diagnosis of cancer in this study). Individual items are scored on a 4-point scale, yielding a total score of 0 to 75, with high scores indicating more PTSD symptoms. In the current study, this single-factor model showed acceptable fit, supporting the validity of using the total score. No consensus exists on cutoff scores for clinically significant levels of PTSD symptoms. However, a total score of 27 or more provided an overall correct classification rate, for traumatic stress, of .80 in a large sample of motor-vehicle-accident survivors including both genders (Coffey, Gudmundsdottir, Beck, Palyo, & Miller, 2006), and has previously been used for cancer (Purnell et al., 2011).

The Illness Perception Questionnaire-Revised (Moss-Morris et al., 2002) was used to assess negative illness perceptions. This questionnaire comprises three parts, the first of which (Identity) asks participants to indicate whether they have experienced any of 15 common symptoms (an additional item of particular relevance to prostate patients—“urinary problems”—was added for this study) since diagnosis and, if so, whether they attribute them to cancer. Items endorsed as having been both experienced and attributed to cancer are counted, providing a total score of 0 to 15. As most patients with early-stage prostate and breast cancer experience few symptoms, this scale was dichotomized (no symptoms vs. 1 or more symptoms). The second part of the Illness Perception Questionnaire-Revised comprises seven cognitive and emotional representation subscales. Items are scored from 1 to 5, with high scores on the Chronic Timeline, Consequences, and Cyclical Timeline subscales indicating a stronger belief that the illness will last a long time, have negative consequences, and be cyclical in nature, respectively, and high scores on the Personal Control, Treatment Control, and Illness Coherence subscales indicating a stronger belief in the controllability of the illness and a greater personal understanding of it, respectively. As this measure was included to assess patients’ cognitive representations, the emotional representation subscale was discarded. The final part, in which items are also scored 1 to 5, measures patients’ causal attributions about their illness. Previously, only psychological and/or behavioral attributions have contributed to the variance explained in quality of life (Scharloo et al., 2010) or emotional distress (Kulik & Kronfeld, 2005; Traeger et al., 2009) after diagnosis of cancer. Therefore, for this study, the seven items that reflect these attributions (i.e., “my own behavior,” “my mental attitude,” “stress or worry,” “my emotional state,” “my personality,” “family problems or worries,” and “overwork”) were used to generate a composite scale (Psychological Cause), and the rest of the items were discarded.

Metacognitive beliefs were measured using the Metacognitions Questionnaire 30 (MCQ-30; Wells & Cartwright-Hatton, 2004). The MCQ-30 was developed specifically to assess key components of the metacognitive model of emotional disorder. It comprises five subscales: Positive Beliefs About Worry, Negative Beliefs About the Danger and Uncontrollability of Worry, Cognitive Confidence, Need to Control Thoughts, and Cognitive Self-Consciousness. The validity of this five-factor model was supported using the current study data. However, as the focus of this study was on testing specific predictions about the relationship of positive and negative metacognitive beliefs about worry with emotional distress, the latter three subscales were discarded. For each subscale of the MCQ-30, items are scored from 1 to 4, yielding total scores of 6 to 24. Participants are asked to indicate how much they generally agree with statements such as “Worrying helps me cope” (Positive Beliefs About Worry subscale) and “My worrying is dangerous for me” (Negative Beliefs About Worry subscale). High scores indicate more positive and negative beliefs about worry, respectively.

Worry was measured using the Penn State Worry Questionnaire (PSWQ; Meyer, Miller, Metzger, & Borkovec, 1990). The PSWQ is a well-established measure developed to assess the level of worry independent of worry content. Participants are asked to rate to what extent statements, such as “When I am under pressure I worry a lot” are “typical of me.” Sixteen items are scored from 1 to 5, yielding a total score of 16 to 80, with higher scores indicating greater worry. However, a single-factor model fit the study data poorly. Some previous studies have indicated a two-factor model (Fresco, Heimberg, Mennin, & Turk, 2002; Yilmaz, Gençöz, & Wells, 2008), with positively (PSWQ +ve) and negatively (PSWQ −ve) phrased items loading on separate factors. This model (with the exception of Item 10, “I never worry about anything,” which loaded on both factors) provided the best fit to the study data and was therefore used in the present study, with Item 10 allowed to cross-load.

The Cognitive Attentional Syndrome Scale (CAS-I; Wells, 2009) was included as an alternative to the PSWQ. Developed primarily as a clinical tool, it is a state measure comprising two distinct parts. The first eight items, scored on a scale from 0 to 8, assess CAS processes and the extent to which individuals have been using maladaptive strategies to cope with negative thoughts or feelings. The second eight items assess metacognitive beliefs about the CAS and were redundant in this study due to inclusion of the MCQ-30. Good internal consistency and significant positive correlations with measures of depression, anxiety, and stress have been reported for the CAS-I scale as a whole (Fergus, Bardeen, & Orcutt, 2012). For the present study, preliminary exploratory factor analysis of the first eight items indicated that a three-factor model provided the best fit. Items 1 (“How much time in the last week have you found yourself dwelling on or worrying about your problems?”) and 2 (“How much time in the last week have you been focusing attention on the things you find threatening [e.g., symptoms, thoughts, danger]?”) loaded on the first factor and were summed to provide an alternative measure of the frequency of worry, with the remaining items being disregarded.

The Medical Outcomes Study MOS social support survey (Sherbourne & Stewart, 1991) controlled for potential effects of perceived emotional support on distress. This 19-item self-report measure was designed to assess four separate dimensions of perceived support among patients with chronic conditions. However, for this study, only the subscales concerning emotional support (Emotional/Informational Support, Positive Social Interaction, and Affectionate Support) were used to produce a total score for “perceived emotional support.” As in a previous study on breast cancer (Hill et al., 2011), this score was dichotomized by designating the patients in the lowest third as having low emotional support.

### Procedure

Clinical staff identified suitable participants, who then received recruitment letters and information sheets before their pretreatment consultations. When patients attended the clinic, those willing to see the researcher were given further information and asked for written consent. Consenting patients completed the questionnaire in the clinic on a handheld PC or on paper, as preferred. Those with insufficient time were given the questionnaire (paper version) to complete at home and return in a paid reply envelope.

### Data Analysis

The data were analyzed using SPSS Version 20, Stata 9, and Mplus v6.12. As fewer than 2% were missing at the scale level, and these data were confirmed to be missing completely at random, missing scores were imputed using the SPSS Expectation-Maximization algorithm (Little & Rubin, 1987). As not all scales were normally distributed, this study used nonparametric statistics or bootstrapping techniques to ensure findings were robust.

Nonparametric statistics (Mann-Whitney or Kruskal-Wallis) were used to compare outcomes by age group (dichotomized at the median), gender, educational level, perceived emotional social support, and stage of disease. When significant differences were found, these variables were entered as covariates in the subsequent analyses.

Preliminary regression analyses were used to identify the illness perceptions associated with each outcome (anxiety, depression, and PTSD symptoms), after controlling for covariates.

**Figure 1 fig1:**
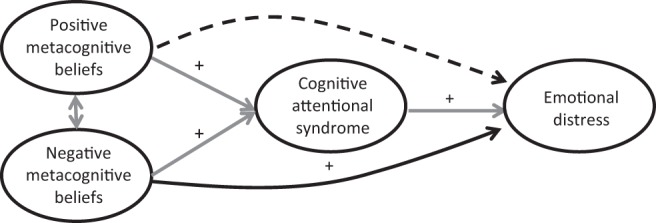
Hypothesized path model of the relationship between metacognitive beliefs and emotional distress. Solid lines are predicted to be significant; dotted lines are not significant; “+” indicates positive direction of effect.

To test the first prediction from the S-REF model, separate hierarchical multiple regression analyses first tested the association of each outcome with metacognitive beliefs, after controlling for identified covariates. These analyses were then repeated, also controlling for the illness perceptions found in preliminary regression analysis to be associated with that outcome. To control for nonnormality, final regression models were robustly assessed using bootstrapped sampling in Stata 9. To test the second prediction from the S-REF model, the data were fitted to the hypothesized model (see Figure 1) using structural equation modeling (SEM) in Mplus Version 6.12 (L. K. Muthén & Muthén, 1998–2010). Because visual inspection suggests there are similarities between some items on the PSWQ and the MCQ-30 subscale Negative Beliefs About Worry, a second model substituting the CAS-I for the PSWQ was included as an additional test to guard against bias due to common method variance. Fit was assessed using the robust weighted least squares estimator (B. Muthén, 1984; B. Muthén, du Toit, & Spesic, 1997) recommended for ordinal categorical data (Brown, 2006). Analyses controlled for identified covariates and were conducted initially using the PSWQ, then repeated using the CAS-I. Adequacy of model fit was assessed based on two incremental fit indices—the Comparative Fit Index (CFI) and the Tucker-Lewis Index (TLI), with values close to .95 indicating a well-fitting model (Hu & Bentler, 1999)—and two absolute misfit indices—the root mean square error of approximation (RMSEA), with values <.05 indicating good fit and 0.5 to .08 indicating adequate fit (Browne & Cudeck, 1993), and the weighted root mean square residual (WRMR), with a cutoff value of .95 indicating good fit (Yu, 2002). For each model, we first confirmed the fit of the measurement component by simultaneously fitting the confirmatory factor analysis (CFA) measurement models for all the included latent variables, allowing them to correlate. The data were then fitted to the structural component of each model to assess the direct and indirect paths linking positive and negative metacognitive beliefs to emotional distress.[Fig-anchor fig1]

## Results

Of 229 participants who completed the questionnaire, 150 were females with breast cancer and 79 were males with prostate cancer. Sample characteristics are summarized in Table 1. A large proportion exceeded cutoff scores for clinically significant anxiety (51%) or PTSD symptoms (59%). Women with breast cancer were more anxious (*U* = 3722, *p* < .001, *r* = −0.31) and reported more PTSD symptoms (*U* = 4105.5, *p* < .001, *r* = −0.25) than men with prostate cancer. Younger patients also reported more anxiety (*U* = 5117, *p* = .004, *r* = −0.19), depression (*U* = 5370, *p* = .017, *r* = −0.16), and PTSD symptoms (*U* = 5238, *p* = .009, *r* = −0.17). However, no outcome was related to education, perceived emotional support, or tumor grade. Therefore, age and gender were the only covariates entered in subsequent analyses.[Table-anchor tbl1]

Results of the preliminary regression analyses are summarized in Table 2. For anxiety and depression, the final model accounted for 32% and 19% of the variance, respectively. After controlling for age and gender, illness perceptions—specifically, higher scores on Identity, Chronic Timeline, Consequences (for anxiety and depression), and Psychological Causes (for anxiety)—explained an additional 20% and 18% of the variance, respectively. In the analysis of PTSD symptoms, the final model accounted for 34% of the variance. Higher scores on the same four illness perception scales, together with higher scores on Treatment Control and lower scores on Illness Coherence, explained an additional 22% of the variance in PTSD symptoms after controlling for age and gender. These findings were confirmed as robust using bootstrapped sampling.[Table-anchor tbl2]

### The Association of Metacognitive Beliefs and Distress

Results of the regression analyses are shown in Table 3. After controlling for age and gender, metacognitive beliefs explained 34% additional variance in anxiety and 14% in depression. Even after controlling also for illness perceptions, metacognitive beliefs added a further 23% and 9% in each outcome, respectively. The final model for anxiety accounted for 52% of the variance. Both the Positive Beliefs About Worry subscale and the Negative Beliefs About Worry subscale made significant individual contributions, with Negative Beliefs About Worry making the largest contribution of all the predictors entered. The final model for depression accounted for 25% of the variance, with the Negative Beliefs About Worry subscale making the largest contribution. Analysis of PTSD symptoms showed a similar pattern (see Table 3). Metacognitive beliefs explained 29% additional variance after controlling for age and gender, and 17% after controlling also for illness perceptions. The final model explained 51% of the variance, with the Negative Beliefs About Worry subscale again making the biggest contribution.[Table-anchor tbl3]

These findings, confirmed as robust using bootstrapped sampling, support the first prediction from the S-REF model that metacognitive beliefs add to the variance explained in distress and trauma after controlling for illness perceptions, with negative beliefs about worry making the biggest contribution to the variance in each outcome.

### SEM of the Relationship Between Metacognitive Beliefs and Emotional Distress

**Figure 2 fig2:**
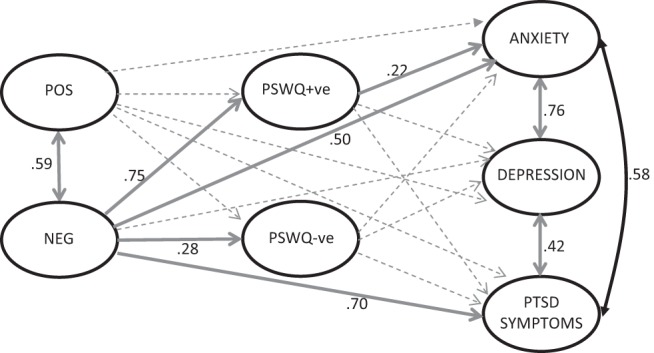
Final path model of relationship of positive and negative metacognitive beliefs with anxiety, depression, and posttraumatic stress disorder symptoms, including mediation by worry (Penn State Worry Questionnaire [PSWQ]). Solid lines, *p* < .05, with standardized coefficients; dotted lines are not significant. Measurement model component of full structural equation model and pathways for covariates (age and gender) is not shown but is available on request from corresponding author. Metacognitions Questionnaire 30 subscales: Positive Beliefs About Worry (POS); Negative Beliefs About Worry (NEG). PSWQ subscales: positively phrased items (PSWQ +ve); negatively phrased (PSWQ −ve).

Confirmatory factor analysis confirmed an excellent fit of the data to the measurement model (see online supplemental materials for further details). The data were then fitted to the full latent variable model, initially using the PSWQ to indicate the putative mediating variable. Age and gender were controlled for within the model (being correlated with the independent variable(s) by default, and having specified causal effects on the putative mediator(s) and final outcome(s). The final path model for anxiety, depression, and PTSD symptoms is shown in Figure 2. The model was a good fit, χ^2^(*df* = 1617) = 1922, *p* < .001, RMSEA = .029 (90% confidence interval [CI] [.02, .03]), CFI/TLI = .98/.98, WRMR = .89). As predicted, significant direct effects were apparent from the Negative Beliefs About Worry subscale to anxiety (β = .50, *p* < .001) and PTSD symptoms (β = .70, *p* < .001), but not from the Positive Beliefs About Worry subscale. In addition, there was a significant indirect path from the Negative Beliefs About Worry subscale to anxiety (β = .16, *p* = .025), mediated by PSWQ +ve, as predicted. However, there were no significant direct or indirect paths from the Negative Beliefs About Worry subscale to depression, and no indirect path mediated by worry to PTSD symptoms. In addition, the paths from the Positive Beliefs About Worry subscale to both PSWQ +ve and PSWQ −ve were not significant.[Fig-anchor fig2]

**Figure 3 fig3:**
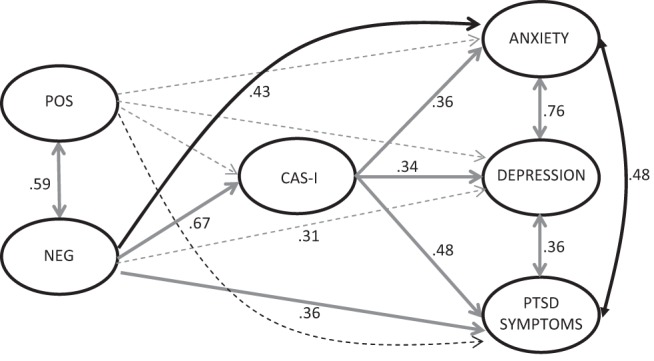
Final path model of relationship between positive and negative metacognitive beliefs and anxiety, depression, and trauma mediated by the CAS-I. Solid lines, *p* < .05, with standardized coefficients; dotted lines are not significant. Measurement model component of full structural equation model and pathways for covariates (age and gender) is not shown but is available on request from corresponding author. Metacognitions Questionnaire 30 subscales: Positive Beliefs About Worry (POS); Negative Beliefs About Worry (NEG).

The model testing was then repeated using the CAS-I subscale as the mediating variable instead of the PSWQ. The final path model is shown in Figure 3. The model was a good fit, χ^2^(*df* = 919) = 1189, *p* < .001, RMSEA = .037 (90% CI [.03, .04]), CFI/TLI = .98/.97, WRMR = .91. The pattern of significant direct paths seen above was replicated; there were significant direct effects of Negative Beliefs About Worry on anxiety (β = .43, *p* < .001) and PTSD symptoms (β = .36, *p* < .001). In addition, there was also a significant indirect effect via the CAS-I on all three outcomes (indirect effects: anxiety, β = .24, *p* < .001; depression, β = .22, *p* = .017; PTSD symptoms, β = .32 *p* < .001). There was no effect of the Positive Beliefs About Worry subscale on either the CAS-I or any of the outcomes.[Fig-anchor fig3]

## Discussion

This is the first study to explore the utility of the S-REF model in an adult cancer population, and, although only cross-sectional, findings are largely consistent with the theory that metacognitive beliefs and perseverative thinking (worry), rather than specific illness perceptions, cause and maintain emotional distress.

### The Relationship Between Metacognitive Beliefs and Distress

Negative illness perceptions were associated with distress after cancer diagnosis, consistent with both the CSM and S-REF models. However, after controlling for age and gender, metacognitive beliefs could explain more of the remaining variance than could illness perceptions for two of the three study outcomes (anxiety, 34% vs. 20%; PTSD symptoms, 29% vs. 22%). In addition, after controlling for age, gender, and illness perceptions, metacognitive beliefs added significantly to the variance in anxiety, depression, and PTSD symptoms, while, in each case, the Negative Beliefs About Worry made the biggest individual contribution to the variance out of all of the predictors. These latter findings are consistent with the S-REF model and with results of previous studies in mental health populations (see Wells, 2009, for a review), the general population (Spada, Mohiyeddini, & Wells, 2008), and Parkinson’s disease patients (Allott et al., 2005), in which the Negative Beliefs About Worry subscale was the predominant contributor to the variance in anxiety and depression.

The regression analysis also indicated that a second set of metacognitive beliefs, the Positive Beliefs About Worry subscale, made a unique contribution to the variance in anxiety. This finding is consistent with the metacognitive model of generalized anxiety disorder (Wells, 1995), in which positive metacognitive beliefs guide the selection of worry as an effective coping strategy, which, in turn, increases emotional distress.

### Mediation of the Relationship Between Metacognitive Beliefs and Distress by the CAS

The S-REF model proposes that the causal link between metacognitive beliefs and distress is the CAS, and, in this respect, the findings partially support predictions from the model. Specifically, the relationship of anxiety with Negative Beliefs About Worry was partially mediated, as predicted, by the PSWQ, and the relationship of all three emotional distress outcomes with the Negative Beliefs About Worry subscale was partially mediated by the CAS-1. That is, the findings are broadly consistent with the theory that negative metacognitive beliefs (e.g., “worry is uncontrollable and dangerous”) cause a direct emotional response (anxiety and trauma symptoms), while also further increasing distress by exacerbating worry and activating metaworry (e.g., “I worry too much about worrying”). The absence of any direct effect of the Negative Beliefs About Worry subscale on depression may reflect the wording of this measure, which focuses specifically on beliefs about worry as opposed to other forms of persistent thinking (i.e., rumination) that are more closely associated with depression.

The hypothesis of full mediation between positive metacognitive beliefs and emotional distress—that is, that positive beliefs about worry, such as “worrying will help me notice if my cancer recurs,” causes emotional distress by driving worry about recurrence and self-focused attention—was not supported. However, S-REF theory would predict that, although positive metacognitive beliefs initially guide an individual toward the selection of CAS processes (i.e., worry) in response to negative thoughts or feelings, it is the negative metacognitive beliefs that “turbo charge” distress by then exacerbating and maintaining these processes. Thus, it is possible that, in a SEM that simultaneously tests the pathways between both sets of metacognitive beliefs and emotional distress, the indirect pathway from the Positive Beliefs About Worry subscale to emotional distress via the CAS is masked by inclusion of Negative Beliefs About Worry.

## Study Implications, Limitations, and Conclusions

In summary, the findings support predictions from the S-REF model that negative metacognitive beliefs cause and maintain distress by activating the CAS. However, because the study was cross-sectional, causality cannot be assumed; maladaptive metacognition may be a consequence of emotional distress, not a cause, and, as these two opposing models would be mathematically equivalent, SEM would be unable to distinguish between them. Therefore, a prospective test of the model is necessary in order to establish temporal precedence of maladaptive metacognition to persistent distress as more compelling evidence of causation. Furthermore, as the SEM was based on the assumption of no hidden confounders, the potential influence of unmeasured common causes cannot be eliminated. In particular, the information available from patients at the time of assessment did not include their history of anxiety, depression, or PTSD symptoms. Consequently, it is possible that, rather than maladaptive metacognitions causing elevated emotional distress, both are consequences of a premorbid psychiatric history. Another limitation is the sample. To balance the competing demands of maximizing recruitment and generalizability, while minimizing prognostic variability, sampling was restricted to the largest tumor groups in each gender—breast and prostate cancer; it cannot be assumed that findings would generalize to other cancers, particularly those with poorer prognosis. Although we controlled for gender (and therefore type of tumor) in the analyses, the study was insufficiently powered for subgroup analyses. Further studies will be needed to test the stability of association of metacognitive beliefs with emotional distress across different tumor populations.

Despite these limitations, this study provides the first evidence of the applicability of the S-REF model to understanding emotional distress and trauma after diagnosis of cancer. Therefore, we suggest that there is potential to reduce vulnerability to emotional distress and trauma by modifying metacognitive beliefs and processes rather than using more traditional cognitive therapies. In a cancer context, an important potential advantage of this metacognitive approach to therapy is that it does not require engagement with the content of negative thoughts about cancer, which many individuals can find difficult or distressing (Baker et al., 2013). However, in order to explore this potential more fully, further study, both prospective and experimental, is warranted.

## Supplementary Material

10.1037/hea0000096.supp

## Figures and Tables

**Table 1 tbl1:** Sample Characteristics (N = 229)

Age	
Mean (*SD*)	61.3 (8.9)
Range	38 to 85
	*n* (% of total *N*)
Gender	
Female	150 (66)
Male	79 (34)
Ethnicity	
White Caucasian	224 (98)
Other	5 (2)
Marital status	
Married/cohabiting	151 (66)
Live alone	46 (20)
Education	
Left school without any qualifications	88 (38)
School qualifications or higher	132 (58)
Employment	
Employed (full/part time)	88 (38)
Retired	99 (43)
Retired (health)	16 (7)
Homemaker	13 (6)
Unemployed	10 (4)
Cancer diagnosis	
Breast	150 (66)
Prostate	79 (34)
Tumor grade	
Low	56 (24)
Intermediate	107 (47)
High	62 (27)
Distress outcomes	
Anxiety (HADS-A >7)	117 (51)
Depression (HADS-D >7)	28 (12)
PTSD symptoms (IES total ≥27)	136 (59)
*Note.* Missing data: marital status, *n* = 5; live alone, *n* = 3; education, *n* = 9; employment, *n* = 3; tumor grade, *n* = 4. HADS-(A/D) = Hospital Anxiety and Depression Scale-(Anxiety/Depression); PTSD = posttraumatic stress disorder; IES = Impact of Events Scale.	

**Table 2 tbl2:** Final Models of the Variance in Anxiety, Depression, and Trauma Explained by Illness Perceptions, After Controlling for Age and Gender

	Anxiety model				Depression model				PTSD symptoms model			
	*R*^2^ change	Beta	*t*	Sig	*R*^2^ change	Beta	*t*	Sig	*R*^2^ change	Beta	*t*	Sig
Constant			−.56	.582			−.79	.428			−.75	.453
Gender	.12*	−.33	−5.49	<.001	.01	−.15	−2.21	.028	.12*	−.27	−4.44	<.001
Age		−.06	−.98	.326		.02	.28	.778		−.15	−2.45	.015
IPQ-R	.20*				.18*				.22*			
Identity (0/1)		.14	2.34	.020		.14	2.10	.037		.18	3.03	.003
Chronic timeline		.17	2.20	.029		.18	2.11	.036		.16	2.11	.036
Cyclical timeline		.12	1.82	.070		.10	1.46	.15		.02	.27	.786
Consequences		.14	2.05	.041		.15	2.00	.046		.17	2.41	.017
Personal control		−.07	−1.25	.212		−.13	−1.96	.051		−.03	−.53	.600
Treatment control		.13	1.71	.088		.07	.82	.412		.19	2.63	.009
Illness coherence		−.00	−.06	.951		−.01	−.17	.865		−.20	−3.16	.002
Psychological cause		.22	3.45	.001		.10	1.42	.156		.16	2.61	.010
Model summary												
*R*^2^	32				.19				.34			
Adj *R*^2^	28				.15				.31			
*Note.* PTSD = posttraumatic stress disorder; Sig = significance; IPQ-R = Illness Perception Questionnaire-Revised; Adj = adjusted.												
* *p* < .001.												

**Table 3 tbl3:** Final Models of the Variance in Anxiety, Depression, and PTSD Symptoms Explained by Metacognitive Beliefs After Controlling for Age and Gender (Model 1) and Age, Gender, and Illness Perceptions (Model 2)

	Anxiety Model 1				Anxiety Model 2			
	*R*^2^ change	Beta	*t*	Sig	*R*^2^ change	Beta	*t*	Sig
Constant			1.20	.233			−1.19	.235
Gender	.12*	−.22	−4.38	<.001	.12*	−.27	−5.34	<.001
Age		−.05	−1.01	.312		−.02	−.48	.629
IPQ-R					.17*			
Identity (0/1)						.14	2.86	.005
Chronic timeline						.10	1.85	.065
Consequences						.07	1.24	.216
Psychological cause						.10	2.09	.038
MCQ-30	.34*				.23*			
POS		.15	2.70	.007		.15	2.75	.006
NEG		.52	9.13	.001		.44	7.92	<.001
Model summary								
*R*^2^	.46				.52			
Adj *R*^2^	.45				.51			
	Depression Model 1				Depression Model 2			
Constant			−.12	.903			−2.31	.022
Gender	.02	−.05	−.81	.417	.02	−.12	−1.87	.064
Age		.00	.02	.983		.05	.74	.458
IPQ-R					.14*			
Identity (0/1)						.14	2.25	.026
Chronic timeline						.17	2.54	.012
Consequences						.11	1.59	.113
MCQ-30	.14*				.09*			
POS		.06	.82	.411		.06	.86	.391
NEG		.36	5.09	<.001		.29	.421	<.001
Model summary								
*R*^2^	.16				.25			
Adj *R*^2^	.14				.22			
	PTSD symptoms Model 1				PTSD symptoms Model 2			
Constant			3.07	.002			.33	.740
Gender	.12*	−.15	−2.90	.004	.12*	−.20	−3.77	<.001
Age		−.15	−2.77	.006		−.11	−2.05	.041
IPQ-R					.22*			
Identity (0/1)						.17	3.28	.001
Chronic timeline						.09	1.30	.194
Consequences						.12	2.02	.045
Treatment control						.10	1.56	.122
Illness coherence						−.16	−2.95	.004
Psychological cause						.05	.99	.322
MCQ-30	.29*				.17*			
POS		.12	2.09	.037		.09	1.58	.115
NEG		.49	8.25	<.001		.41	7.14	<.001
Model summary								
*R*^2^	.41				.51			
Adj *R*^2^	.40				.48			
*Note.* PTSD = posttraumatic stress disorder; Sig = significance; IPQ-R = Illness Perception Questionnaire-Revised; MCQ-30 = Metacognitions Questionnaire 30; POS = Positive Beliefs About Worry subscale; NEG = Negative Beliefs About Worry subscale; Adj = adjusted.								
* *p* < .001.
