# Supplementary material for: The Association of Metacognitive Beliefs With Emotional Distress After Diagnosis of Cancer
Source: Health Psychol. 2014 Aug 18;34(3):207–15. doi: 10.1037/hea0000096 (PMC4321533; doi:10.1037/hea0000096)
Supplement: Supplementary file 1 [file hea1287MCT1HPAddSuppjan14.docx]

**Additional Supplemental Material**

**Appendix 1: Fit Statistics and standardized factors loadings for the CFA measurement model of positive and negative metacognitive beliefs, anxiety, depression, PTSD symptoms and worry (PSWQ)**

**Chi-Square Test of Model Fit** = 1805.639* (DF 1517), P-Value p<0.001

**RMSEA** = 0.029 (90% C.I . = 0.024 – 0.035), Probability RMSEA <= .05 = 1.000

**CFI/TLI** = 0.980/.979

**WRMR** = 0.89

**Table A1: CFA Standardized model results**

|  | **Estimate** | **S.E** |
| --- | --- | --- |
| **POS BY** |  |  |
| MCQ1 | 0.758 | 0.062 |
| MCQ7 | 0.860 | 0.042 |
| MCQ10 | 0.861 | 0.033 |
| MCQ19 | 0.922 | 0.029 |
| MCQ23 | 0.826 | 0.034 |
| MCQ28 | 0.889 | 0.033 |
| **NEG BY** |  |  |
| MCQ2 | 0.443 | 0.066 |
| MCQ4 | 0.672 | 0.052 |
| MCQ9 | 0.829 | 0.031 |
| MCQ11 | 0.863 | 0.025 |
| MCQ15 | 0.762 | 0.051 |
| MCQ21 | 0.848 | 0.028 |
| **WORRYA BY** |  |  |
| W2 | 0.901 | 0.020 |
| W4 | 0.830 | 0.027 |
| W5 | 0.902 | 0.018 |
| W6 | 0.815 | 0.027 |
| W7 | 0.895 | 0.021 |
| W9 | 0.726 | 0.039 |
| W10 | 0.377 | 0.053 |
| W12 | 0.847 | 0.025 |
| W13 | 0.875 | 0.021 |
| W14 | 0.919 | 0.016 |
| W15 | 0.892 | 0.023 |
| W16 | 0.696 | 0.038 |
| **WORRYB BY** |  |  |
| W1 | 0.446 | 0.110 |
| W3 | 0.516 | 0.098 |
| W8 | 0.753 | 0.103 |
| W10 | 0.542 | 0.056 |
| W11 | 0.687 | 0.093 |
| **ANXIETY BY** |  |  |
| HADS1 | 0.850 | 0.030 |
| HADS4 | 0.763 | 0.037 |
| HADS5 | 0.867 | 0.024 |
| HADS8 | 0.556 | 0.057 |
| HADS9 | 0.831 | 0.033 |
| HADS12 | 0.807 | 0.034 |
| HADS13 | 0.808 | 0.034 |
| **DEPRESS BY** |  |  |
| HADS2 | 0.733 | 0.049 |
| HADS3 | 0.784 | 0.045 |
| HADS6 | 0.604 | 0.080 |
| HADS7 | 0.835 | 0.062 |
| HADS10 | 0.836 | 0.046 |
| HADS11 | 0.795 | 0.051 |
| HADS14 | 0.739 | 0.075 |
| **TRAUMA BY** |  |  |
| IES1 | 0.735 | 0.044 |
| IES2 | 0.524 | 0.062 |
| IES3 | 0.580 | 0.057 |
| IES4 | 0.735 | 0.041 |
| IES5 | 0.830 | 0.032 |
| IES6 | 0.574 | 0.062 |
| IES7 | 0.728 | 0.046 |
| IES8 | 0.674 | 0.048 |
| IES9 | 0.564 | 0.060 |
| IES10 | 0.735 | 0.039 |
| IES11 | 0.746 | 0.039 |
| IES12 | 0.801 | 0.035 |
| IES13 | 0.757 | 0.038 |
| IES14 | 0.831 | 0.029 |
| IES15 | 0.723 | 0.041 |

N.B. All p values were significant at the p<.001 level.

**Appendix 2: Fit Statistics and standardized factors loadings for the CFA measurement model of the relationship between positive and negative metacognitive beliefs, anxiety, depression, PTSD symptoms and frequency of worry (CAS-I )**

**Chi-Square Test of Model Fit** = 1098.013* (DF 845), P-Value p<.001

**RMSEA** = 0.037(90% C.I. = 0.030 - 0.043), Probability RMSEA <= .05 = 1.000

**CFI/TLI** = 0.976/ 0.974

**WRMR** = 0.916

**Table A2: CFA Standardized model results**

|  | **Estimate** | **S.E.** |
| --- | --- | --- |
| POS……BY |  |  |
| MCQ1 | 0.737 | 0.062 |
| MCQ7 | 0.879 | 0.038 |
| MCQ10 | 0.855 | 0.032 |
| MCQ19 | 0.937 | 0.026 |
| MCQ23 | 0.829 | 0.032 |
| MCQ28 | 0.883 | 0.032 |
| NEG BY |  |  |
| MCQ2 | 0.438 | 0.069 |
| MCQ4 | 0.637 | 0.058 |
| MCQ9 | 0.850 | 0.030 |
| MCQ11 | 0.900 | 0.025 |
| MCQ15 | 0.724 | 0.054 |
| MCQ21 | 0.819 | 0.033 |
| CASWORRY BY |  |  |
| CAS1 | 0.937 | 0.018 |
| CAS2 | 0.902 | 0.019 |
| ANXIETY BY |  |  |
| HADS1 | 0.827 | 0.032 |
| HADS4 | 0.777 | 0.035 |
| HADS5 | 0.868 | 0.023 |
| HADS8 | 0.544 | 0.056 |
| HADS9 | 0.850 | 0.029 |
| HADS12 | 0.803 | 0.035 |
| HADS13 | 0.812 | 0.033 |
| DEPRES BY |  |  |
| HADS2 | 0.729 | 0.045 |
| HADS3 | 0.789 | 0.044 |
| HADS6 | 0.598 | 0.076 |
| HADS7 | 0.837 | 0.058 |
| HADS10 | 0.842 | 0.043 |
| HADS11 | 0.795 | 0.048 |
| HADS14 | 0.745 | 0.071 |
| TRAUMA BY |  |  |
| IES1 | 0.769 | 0.036 |
| IES2 | 0.539 | 0.058 |
| IES3 | 0.565 | 0.055 |
| IES4 | 0.745 | 0.039 |
| IES5 | 0.836 | 0.028 |
| IES6 | 0.572 | 0.060 |
| IES7 | 0.710 | 0.046 |
| IES8 | 0.678 | 0.045 |
| IES9 | 0.535 | 0.059 |
| IES10 | 0.741 | 0.037 |
| IES11 | 0.737 | 0.039 |
| IES12 | 0.795 | 0.033 |
| IES13 | 0.737 | 0.038 |
| IES14 | 0.830 | 0.027 |
| IES15 | 0.725 | 0.038 |

N.B. All p values were significant at the p<.001 level.

**Appendix 3: Standardized model results for the SEM of the relationship between positive and negative metacognitive beliefs and anxiety, depression and PTSD symptoms mediated by worry (PSWQ)**

**Table A3: Standardized model results for Fig 2**

|  | **Estimate** | **S.E** | **P-Value** |
| --- | --- | --- | --- |
| Worry+ve …ON |  |  |  |
| POS | 0.118 | 0.059 | 0.045 |
| NEG | 0.753 | 0.049 | <.001 |
| Age | -0.084 | 0,044 | 0.058 |
| Gender | 0.048 | 0.044 | 0.272 |
| Worry-ve…ON |  |  |  |
| POS | 0.139 | 0.097 | 0.153 |
| NEG | 0.281 | 0.097 | 0.004 |
| Age | 0.027 | 0.078 | 0.731 |
| Gender | -0.111 | 0.074 | 0.134 |
| Anxiety…ON |  |  |  |
| POS | 0.070 | 0.076 | 0.356 |
| NEG | 0.504 | 0.101 | <.001 |
| Worry+ve | 0.218 | 0.096 | 0.024 |
| Worry-ve | 0.003 | 0.053 | 0.958 |
| Age | 0.012 | 0.049 | 0.813 |
| Gender | 0.219 | 0.048 | <.001 |
| Depression…ON |  |  |  |
| POS | 0.019 | 0.110 | 0.864 |
| NEG | 0.219 | 0.162 | 0.174 |
| Worry+ve | 0.243 | 0.147 | 0.099 |
| Worry-ve | 0.048 | 0.080 | 0.546 |
| Age | 0.012 | 0.061 | 0.840 |
| Gender | 0.041 | 0.070 | 0.553 |
| PTSD symptoms... ON |  |  |  |
| POS | 0.058 | 0.074 | 0.437 |
| NEG | 0.701 | 0.118 | <.001 |
| Worry+ve | -0.040 | 0.100 | 0.692 |
| Worry-ve | 0.020 | 0.051 | 0.699 |
| Age | -0.071 | 0.055 | 0.196 |
| Gender | 0.144 | 0.053 | 0.006 |

**Appendix 4: Standardized model results for the SEM of the relationship between positive and negative metacognitive beliefs and anxiety, depression and PTSD symptoms mediated by frequency of worry (CAS-I)**

**Table A4: Standardized model results for Fig 3**

|  | **Estimate** | **S.E** | **P-Value** |
| --- | --- | --- | --- |
| CAS-I …ON |  |  |  |
| POS | 0.169 | 0.085 | 0.046 |
| NEG | 0.666 | 0.071 | <.001 |
| Age | -0.054 | 0.053 | 0.310 |
| Gender | 0.100 | 0.047 | 0.035 |
| Anxiety…ON |  |  |  |
| POS | 0.035 | 0.084 | 0.679 |
| NEG | 0.426 | 0.092 | <.001 |
| CAS-I | 0.364 | 0.095 | <.001 |
| Age | 0.012 | 0.049 | 0.800 |
| Gender | 0.188 | 0.049 | <.001 |
| Depression…ON |  |  |  |
| POS | -0.003 | 0.113 | 0.975 |
| NEG | 0.191 | 0.147 | 0.192 |
| CAS-I | 0.337 | 0.133 | 0.012 |
| Age | 0.012 | 0.058 | 0.840 |
| Gender | 0.015 | 0.071 | 0.829 |
| PTSD symptoms... ON |  |  |  |
| POS | -0.022 | 0.087 | 0.803 |
| NEG | 0.357 | 0.101 | <.001 |
| CAS-I | 0.476 | 0.099 | <.001 |
| Age | -0.046 | 0.048 | 0.337 |
| Gender | 0.091 | 0.051 | 0.075 |
